# Phosphatidylinositol 3-kinase p110δ expression in Merkel cell carcinoma

**DOI:** 10.18632/oncotarget.25619

**Published:** 2018-07-03

**Authors:** Emil Chteinberg, Dorit Rennspiess, Ryan Sambo, Samantha Tauchmann, Nicole W.J. Kelleners-Smeets, Véronique Winnepenninckx, Ernst-Jan Speel, Anna Kordelia Kurz, Martin Zenke, Axel zur Hausen

**Affiliations:** ^1^ Department of Pathology, GROW-School for Oncology and Developmental Biology, Maastricht University Medical Centre, Maastricht, The Netherlands; ^2^ Institute for Biomedical Engineering, Department of Cell Biology, RWTH Aachen University Hospital, Aachen, Germany; ^3^ Helmholtz Institute for Biomedical Engineering, RWTH Aachen University, Aachen, Germany; ^4^ Department of Dermatology, GROW-School for Oncology and Developmental Biology, Maastricht University Medical Centre, Maastricht, The Netherlands; ^5^ Department of Internal Medicine IV, RWTH Aachen University Hospital, Aachen, Germany

**Keywords:** Merkel cell carcinoma, phosphatidylinositol 3-kinase, chemotherapeutics

## Abstract

The prognosis of stage III/IV Merkel cell carcinoma (MCC) is very poor. The Phosphatidylinositol 3-kinase p110δ specific inhibitor idelalisib has recently been reported to induce complete clinical remission in a stage IV MCC patient. Here we assessed the expression of p110δ in primary MCC and MCC cell lines including its functionality.

Immunofluorescence microscopy revealed a specific cytoplasmic p110δ expression in 71.4% of the tested MCCs and in all tested MCC cell lines. Compared to the B cell leukemia cell line REH all MCC cell lines, except MKL-1, revealed a lower response towards the treatment with idelalisib. MKL-1 showed a 10-fold higher IC_50_ compared to REH which was accompanied by a significant decrease of Akt phosphorylation. However, treating the MCC cells with the specific PI3K p110α subunit inhibitor BYL719 led to a more effective decrease of the cell viability compared to idelalisib: WaGa cells 30-fold, PeTa cells 15-fold and all other MCC cell lines 3-fold.

Although PI3K p110δ is expressed in the majority of MCCs and cell lines its inhibition by idelalisib alone does not suffice to effectively affect MCC cells viability.

## INTRODUCTION

Merkel cell carcinoma (MCC) is a very aggressive virus associated non-melanoma human skin cancer [1–3]. The Merkel cell polyomavirus (MCPyV) is clonally integrated in more than 80% of the MCCs [4, 5] and it has been shown that the expression of the oncogenic T-antigens are important drivers for the oncogenesis of MCC [6, 7]. Next to MCPyV, UV exposure, age and immune deficiency are contributing to the pathogenesis of MCC [1]. In addition, the risk to develop MCC is significantly increased upon solid organ transplantation (23.8-fold) and for HIV patients (11-fold) [8, 9].

Despite its rarity the incidence of MCC has tripled in the USA and Europe [1]. This and the low 5-year survival rate of 20% of MCCs with distant metastasis emphasizes the need for a unique treatment [10]. Recently, a complete clinical response induced by the phosphoinositide 3-kinase (PI3K) p110δ selective inhibitor idelalisib in a patient with stage IV - with proven p110δ transcript expression by quantitative RT-PCR - has been reported by Shiver *et al.* [11].

The activity of the PI3K-pathway has already been studied in MCC by inhibiting the p110α subunit [12, 13]. Yet, it remains unclear to which extent the transcript expression of PI3K p110δ leads to a significant specific protein expression in MCC and to which extent p110δ contributes to the PI3K pathway activity in MCC. It is expected that elucidating PI3K p110δ activity in MCC might help to identify potential additional therapeutic options for this currently poorly treatable non-melanoma skin cancer.

Here we assessed the expression of p110δ in 21 MCC tissues and 7 MCC cell lines. The functionality of p110δ was analyzed by idelalisib treatment of MCC cell lines and compared to the p110α subunit by treatment with the p110α specific inhibitor BYL719.

## RESULTS

### Expression of PI3K p110δ in MCC

**Figure 1:**
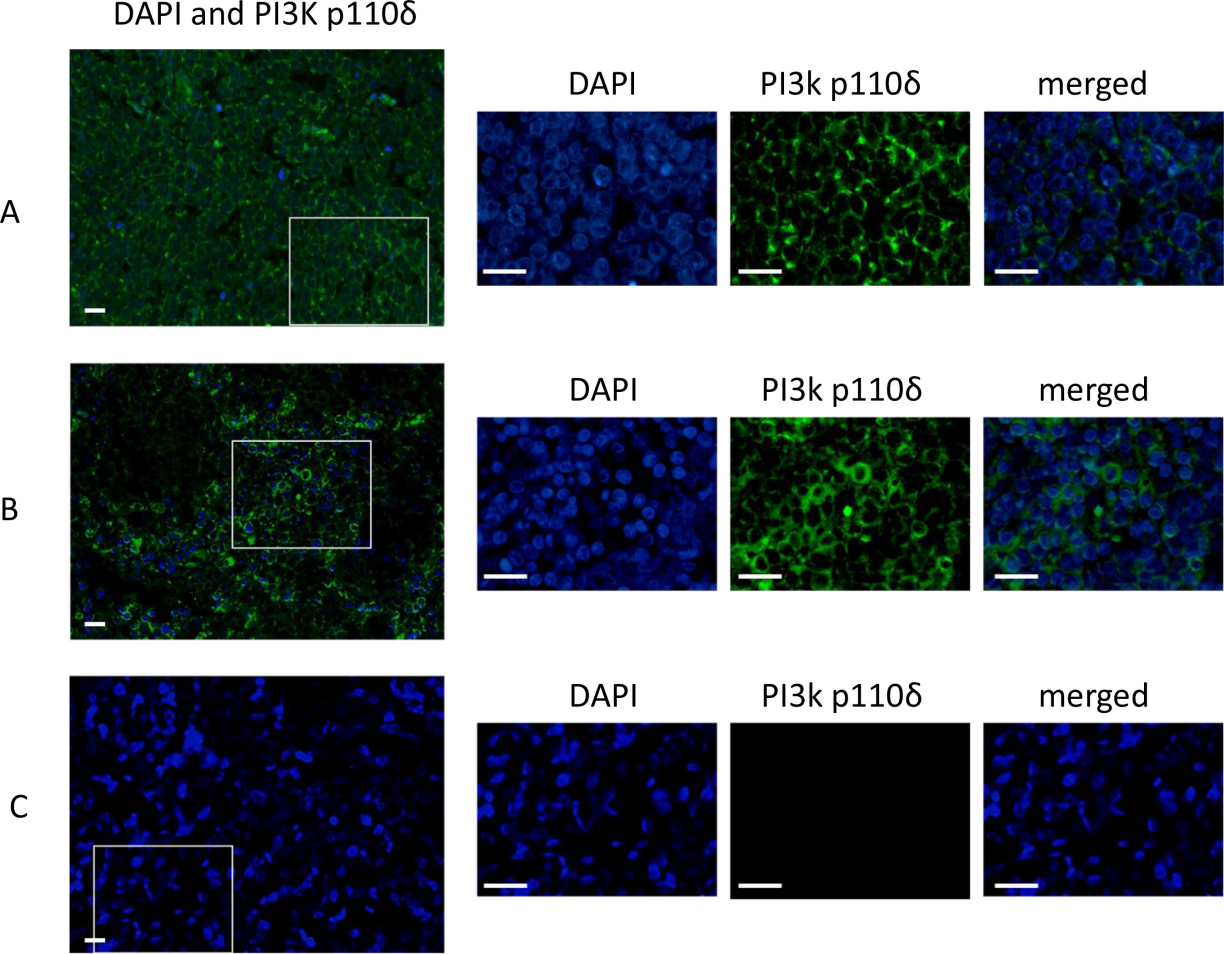
Immunofluorescence staining of PI3K p110δ in MCCs The IFM photographs of three MCCs ID14 (**A**), ID15 (**B**) and ID7 (**C**). The wide merged photographs (DAPI staining and PI3K p110δ) were taken at a magnitude of 10×. The squares in the pictures indicate the region which is chosen for an image enlargement which was taken at a magnitude of 63×. The enlarged pictures show separately the nucleus by the DAPI staining and the PI3K p110δ staining. The merged pictures show a specific cytoplasmic staining of the p110δ subunit in ID14 (A) and ID15 (B). ID7 (C) is an example of a p110 δ negative MCC. The scale bares represent a length of 100 µm.

The expression of PI3K p110δ was analyzed in 18 primary and in three metastatic MCCs by immunofluorescence microscopy (IFM). A specific cytoplasmic staining could be observed in 13 of 18 primary MCC (72.2%) and in 2 out of 3 metastatic MCC (66.6%) (Table 1 and Figure 1). The MCPyV status of all tested MCC tissues was assessed, an example of one MCPyV-positive and one MCPyV-negative tissue is shown in Supplementary Figure 1. 3 out of 18 primary MCCs were negative for MCPyV, which were positive for the p110δ subunit.

**Table 1:** Clinico-pathological data of MCC patients and corresponding tissues including the results of PI3K p110δ expression as tested by immunofluorescence microscopy (IFM)

primary MCCs							
ID	gender	age	location	dx	histo.	MCPyV	PI3K p110δ
**1**	m	63	head	MCC	int.	pos.	+
**2**	m	92	ear	MCC	s.c.	neg.	++
**3**	f	85	buttocks	MCC	s.c.	pos.	++
**4**	m	69	lip	MCC	int.	pos.	++
**5**	f	93	upper eye lid	MCC	int.	pos.	+heterogen.
**6**	f	60	tongue	MCC	int./s.c.	pos.	−
**7**	m	74	upper leg	MCC	int.	pos.	−
**8**	m	93	head	MCC	int.	neg.	++
**9**	f	76	buccal	MCC	int.	pos.	−
**12**	f	83	upper eye lid	MCC	int.	pos.	++
**14**	m	74	upper leg	MCC	int.	pos.	++
**15**	m	77	neck	MCC	int.	neg.	++
**16**	f	66	arm	MCC	int.	pos.	++
**17**	m	79	head	MCC	int.	pos.	−
**19**	m	68	buttocks	MCC	int.	pos.	++
**20**	f	58	buccal	MCC	int./s.c.	pos.	++
**24**	m	74	upper leg	MCC	int.	pos.	−
**30**	m	63	upper lip	MCC	int.	pos.	++

metastases MCC							
ID	gender	age	location	dx	histo.	MCPyV	PI3K p110δ
**21**	m	71	pancreas	MCC met.	int.	pos.	+
**22**	f	75	LN neck	MCC met.	int.	pos.	++
**25**	f	66	upper leg	MCC met.	int.	pos.	−

Abbreviations: ID= identity; m= male; f= female; dx= diagnosis; LN = lymph node; MCC= Merkel cell carcinoma; Histo= histological subtype; int.= intermediate; s. c. = small cell; MCPyV = Merkel cell polyomavirus; IHC = immunohistochemistry; pos.= positive; neg.= negative; heterogen. = heterogeneous; − = no expression; + = weak expression; ++ = moderate expression; +++ = strong expression; met. = metastasis.

### Expression of PI3K p110δ and p110α in MCC cell lines

**Figure 2:**
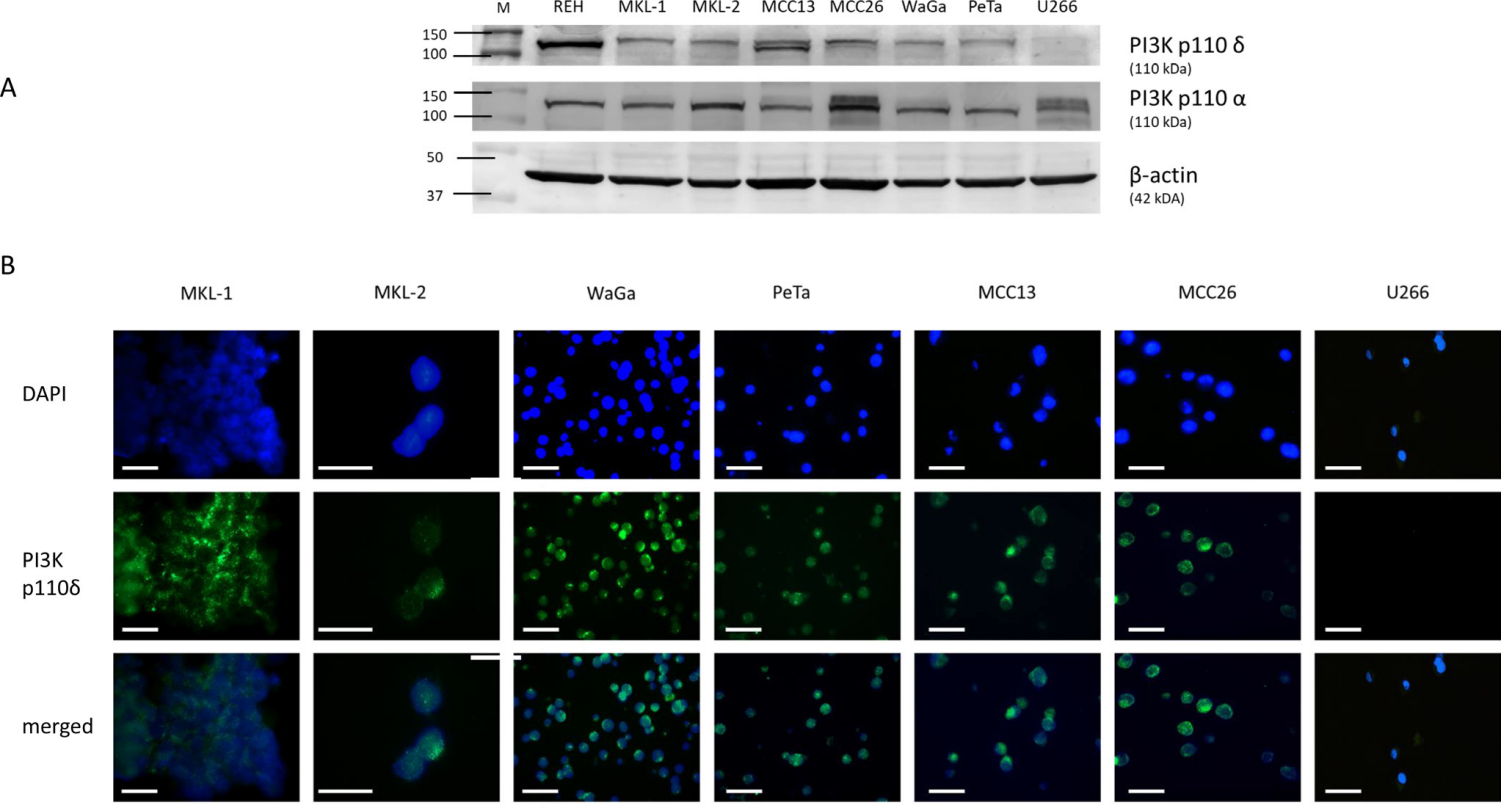
Assessment of PI3K p110δ expression by Western blotting (**A**) and immunofluorescence microscopy (**B**). **(**A) Expression level of PI3K p110δ and PI3K p110α in cell lysates of REH, MKL-1, MKL-2, WaGa, PeTa, MCC13, MCC26, and U266. All cell lines except U266 (PI3K p110δ negative control) revealed a specific PI3K p110δ protein expression at 110 kDa, whereas PI3K p110α expression was detected in all cell lines. The loading control β-actin is located at 42 kDa. (B) The corresponding IFM photographs for DAPI, PI3K p110δ and merged are shown for these cell lines. The merged photograph identifies PI3K p110δ expression within the cytoplasm. According to Figure 2A U266 cells reveal no detectable PI3K p110δ expression by IFM. The photos were taken with 63× magnification. The scale bars represent a length of 100 µm.

In addition, the expression of the PI3K p110α and PI3K p110δ was assessed in the MCC cell lines MKL-1, MKL-2, WaGa, PeTa, MCC13 and MCC26. The B-ALL cell line REH was used as positive control and the human multiple myeloma cell line U266 as negative control (Figure 2A) [14]. A strong protein band at the expected size of PI3K p110δ of 119kDa could be observed for the cell lysate of REH. Thinner protein bands were also visible in the MCPyV-positive and MCPyV-negative cell line lysates and completely absent in U266 cell lysate. In comparison, all cell line lysates showed a strong protein band at 119 kDa specific for PI3K p110α (Figure 2A). The results for the p110δ subunit were confirmed by immunofluorescence microscopy (IFM): in all cell lines except U266 a specific cytoplasmic staining could be detected (Figure 2B). Thus, PI3K p110δ is expressed in the majority of primary and metastatic MCCs and in MCC cell lines irrespective of the MCPyV status.

### Treatment with PI3K inhibitors

**Figure 3:**
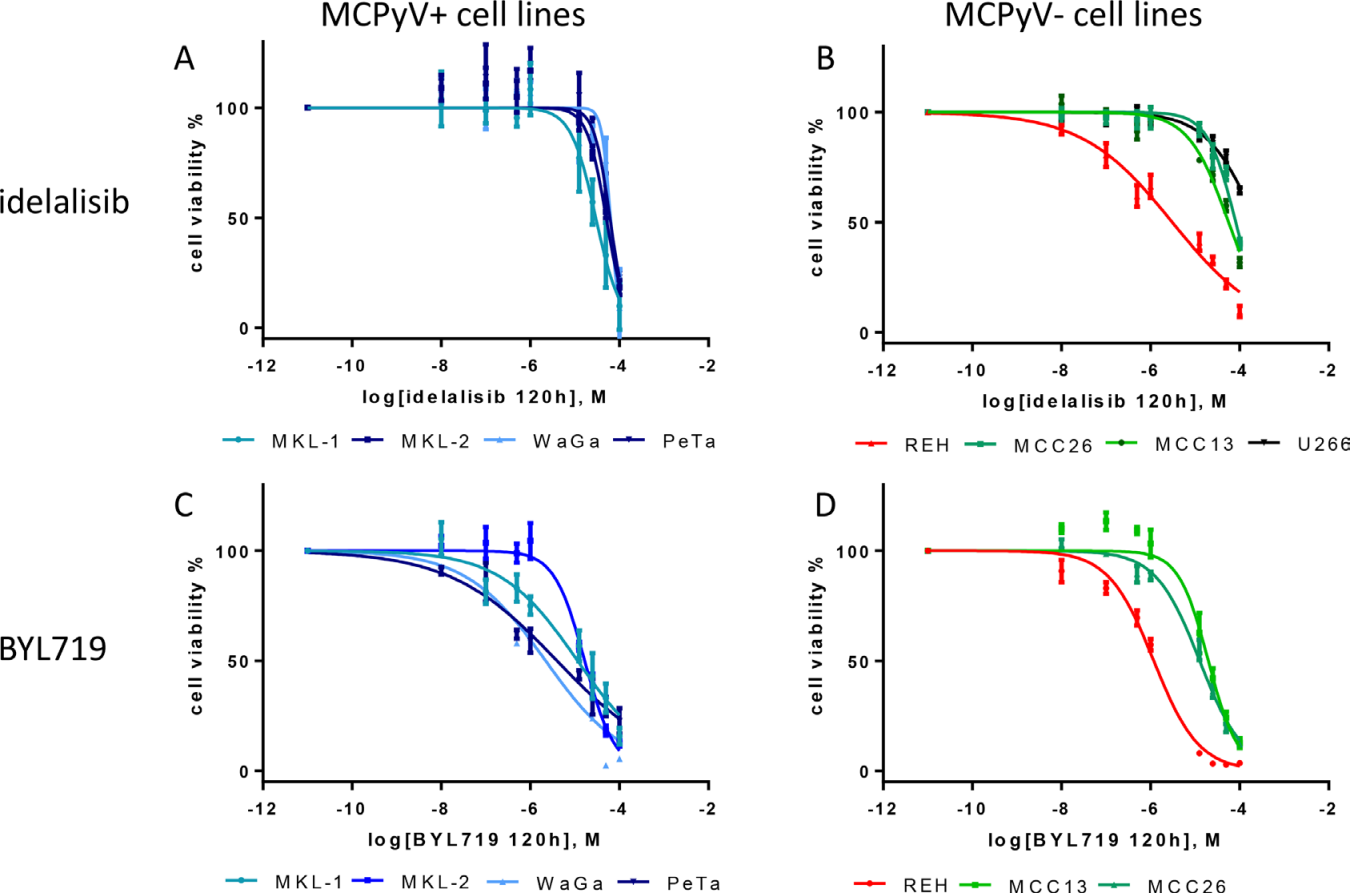
Dose-response curves of the MCPyV-positive MKL-1, MKL-2, WaGa, PeTa (**A** and **C**), and MCPyV-negative MCC13, MCC26, REH and U266 (**B** and **D**) cell lines after 120 h treatment with varying molar concentrations [M] of idelalisib and BYL719. The cell viability is inhibited by idelalisib or BYL719 in all cell lines in a dose-dependent manner. IC_50_ were calculated from 3 independent (*n* = 3) experiments with eight replicates each. Bars symbolize the standard deviation of the mean. The overall *p* values of all dose response curves besided the treatment of U266 are < 0.001.

The cell lines were tested for their sensitivity towards the PI3K p110δ specific inhibitor idelalisib. A decrease of the cell viability of almost all treated cell lines was observed after 120 h incubation. The best response was observed in the B-ALL cell line REH with an IC_50_ of 3.1 µM. The cell lines MKL-2, WaGa, PeTa and MCC13 showed for the idelalisib treatment almost a comparable range of IC_50_ 50 µM to 63 µM. The MKL-1 cells were 2-fold more sensitive compared to all other MCC cell lines. MCC26 cells showed a 1.4- to 1.6-fold weaker response compared to all other MCC cell lines. The multiple myeloma cell line U266 showed a decrease of the cell viability at a concentration of 12.5 µM, an IC_50_ value could not be determined for this PI3K p110δ-negative cell line. In general, all MCC cell lines showed with an overall *p*-value < 0.001 a significant dose-dependent sensitivity to idelalisib after 120 h incubation (Figure 3 and Table 2).

**Table 2:** IC_50_ values of idelalisib and BYL719 treatment of MCC, B-ALL and multiple myeloma cell lines

cell lines	cancer type	MCPyV	IC_50_ idelalisib (µM)	IC_50_ Byl719 (µM)
**MKL-1**	MCC	pos.	29.6	11.5
**MKL-2**	MCC	pos.	50.7	16.6
**WaGa**	MCC	pos.	63.1	2.2
**PeTa**	MCC	pos.	59.2	4.2
**MCC13**	MCC	neg.	56.5	20.0
**MCC26**	MCC	neg.	81.9	12.7
**REH**	B-ALL	neg.	3.1	1.2
**U266**	multiple myeloma	neg.	not deter.	n.d.

Abbreviations: IC_50_= half maximal inhibitory concentration, B-ALL= B cell acute lymphoblastic leukemia pos.= positive; neg.= negative; not. deter. = not determinable adjusted in the manuscript, n.d.= not done.

**Figure 4:**
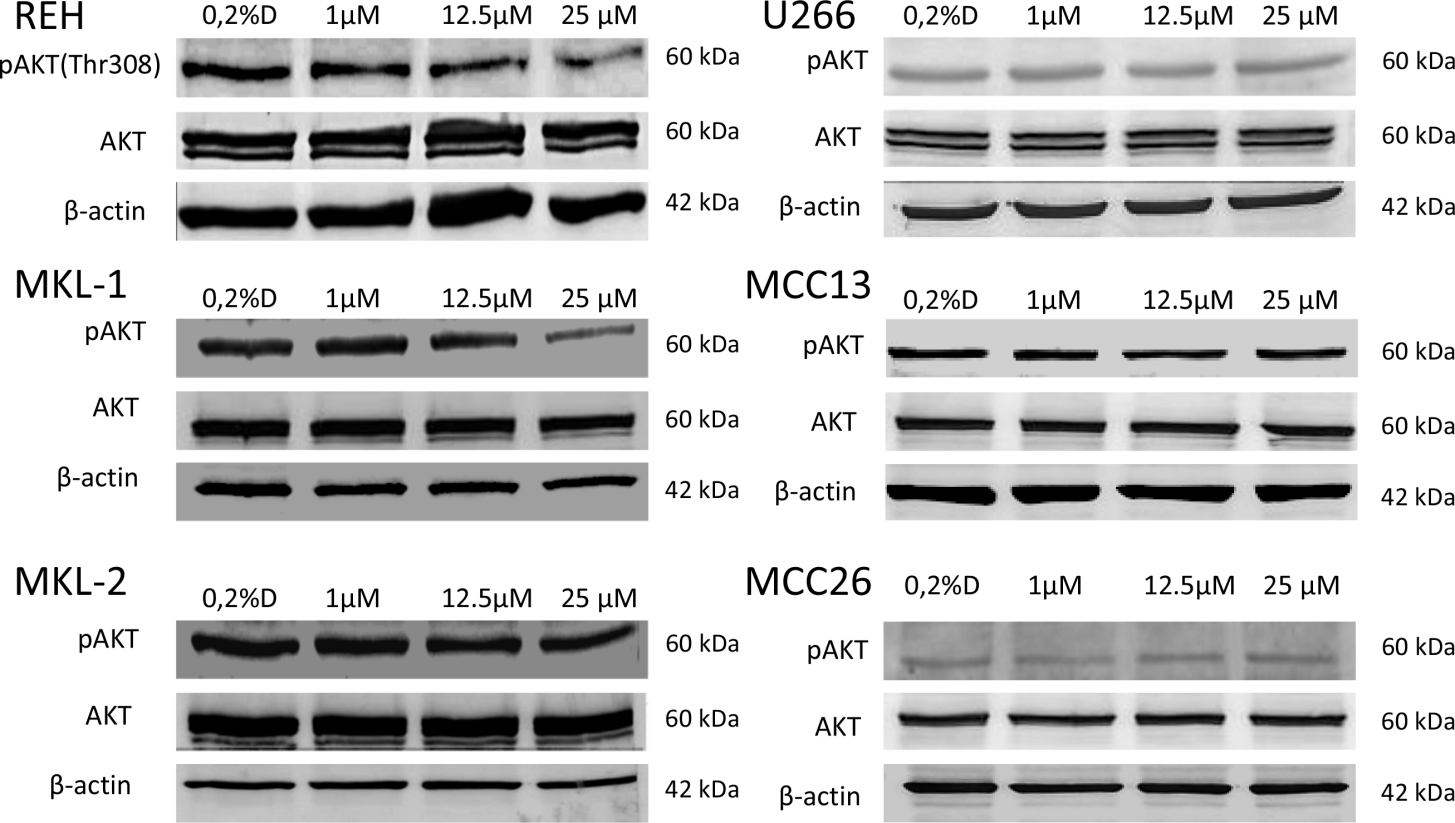
Western blotting of AKT phosphorylation (p-AKT) in idelalisib treated cell lines REH, MKL-1, MKL-2, MCC13, MCC26 and U266 For p-AKT and AKT a t 60 kDa protein band could be detected in all cell lines. Only in the MCC cell line MKL-1 and the B-ALL cell line REH a decrease of p-AKT protein band density with increasing idelalisib concentrations could be observed. AKT and β-actin (42 kDa) was used as a loading control. 0.2% DMSO (0.2%D) without idelalisib were used as a treatment negative control.

The effect of idelalisib on the PI3K pathway of the treated cell lines was tested by Western blotting analyzing the threonine 308 phosphorylation of protein kinase B (AKT) which is located downstream of PI3K. A significant decrease of AKT phosphorylation was restricted to REH and MKL-1 with increasing concentrations of idelalisib (Figure 4).

In addition, the MCC cell lines and the B-ALL cell line REH were treated with the specific PI3K p110α inhibitor BYL719. In general, all cell lines showed a higher sensitivity towards BYL719 treatment compared to the idelalisib treatment. Again, REH showed the strongest response compared to all other cell lines. The MCPyV-positive cell lines WaGa and PeTa were more sensitive for BYL719 compared to MKL-1 and MKL-2 (Figure 3 and Table 2).

## DISCUSSION

Under physiological conditions the PI3K p110δ subunit plays an important role in the differentiation of early B-cells into mature B-cells [15]. In the present study, we tested the expression and functionality of PI3K p110δ in MCC. Using IFM we found that 71% of MCC tested, revealed a specific cytoplasmic expression of the p110δ subunit. Prior to the use of IFM we had checked the monoclonal antibody for its specificity using Western blotting. All MCC cell lines were PI3K p110δ positive by Western blotting as confirmed by IFM. The B-ALL cell line REH revealed the highest protein PI3K p110δ expression compared to the other cell lines. To the best of our knowledge this is the first proof of PI3K p110δ protein expression in MCC tissues and MCC cell lines. Previously, Shiver *et al.* have reported PI3K p110δ expression on the transcript level in one MCC sample of a stage IV MCC patient [11]. This patient was treated with combined radiotherapy and idelalisib which is a selective PI3K p110δ inhibitor, that has effectively been used in the treatment of chronic lymphocytic leukemia (CLL), B-ALL and Hodgkin lymphoma (HL) [16]. In our cell culture treatment assay idelalisib significantly affected the cell viability of all MCC cell lines. However, compared to the B-ALL cell line REH this decrease of the cell viability has to be interpreted as weak.

The results of our analysis of the PI3K downstream pathway could possibly provide an explanation for the weak response of the MCC cell lines towards idelalisib treatment. Since AKT becomes phosphorylated upon PI3K activation we analyzed the phosphorylation status of AKT in the MCC cell lines. Of interest, only MKL-1 showed a decrease of phosphorylated AKT with increasing concentrations of idelalisib. Although this effect was 10-fold weaker as compared to the B-ALL cell line REH, MKL-1 was the only MCC cell line which revealed a comparable effect as seen in REH cells implying that PI3K p110δ is functional in MKL-1.

Functional PI3K p110δ is primarily restricted to leukocytes and has been reported to be critical for the activation, proliferation and survival of B-cells [17] and the differentiation of pre-pro B-cells in pro B-cells [15]. Thus, together with the recently reported pre-pro/pro- B- cell differentiation of MCC, i.e. TdT and PAX-5, immunoglobulin (Ig) expression and Ig rearrangements [18, 19], the functional PI3K p110δ expression in MCC might add another tesserae in the discussion of the cellular origin of MCC.

The inhibition of the PI3K p110α by BYL719 of the same cell lines has revealed a stronger impact on the MCC cell lines. The cell line REH showed again the best response towards the treatment with BYL719, followed by WaGa, PeTa, MKL-1, MKL-2, MCC26 and MCC13. Hafner and colleagues had treated the MCC cell lines WaGa, MKL-1, MKL-2 and MCC13 with another PI3K p110α inhibitor LY-294002 [13]. They report IC_50_ values which are 6-fold, for WaGa, 1.6-fold for MKL-1, 1.8-fold for MKL-2 and 1.5-fold for MCC13 lower as compared to the calculated concentrations for BYL719. This might indicate that BYL719 is either more specific for the alpha subunit or is easier penetrating the cells. However, the order of the response to the drugs is still the same, WaGa cells are most responsive to the treatments followed by MKL-1, MKL-2 and MCC13 [13].

This strong impact on the MCC cell lines is not observed with idelalisib treatment rendering PI3K p110δ expression and/or function less important for cell viability and for the PI3K pathway function of almost all MCC cell lines tested as shown by the unaltered pAKT concentration. Of interest, recent reports have addressed the mechanism of idelalisib resistance in leukemia cells [20–22] and report PI3K p110α upregulating mutations and upregulation of the downstream target in the Src family kinase (SFK) and Wnt pathway as underlying mechanisms for the resistance towards idelalisib treatment. According to the Western blotting results in this study of the p110α and p110δ detection in the treated cell lines, a much higher expression of p110α compared to p110δ is found. In combination with the much more effective treatment with BYL-719 compared to idelalisib, our data indicate that the resistance might also here be based on the higher level of p110α expression compared to p110δ in MCC cell lines.

To what extent the findings of our study add to the understanding of the recent case report of Shiver *et al.* (10) cannot completely be resolved, because the patient described by Shiver *et al.* did also receive radiotherapy. Iyer and colleagues described single-fraction radiation therapy for MCCs as a convenient alternative for chemotherapy [23]. So it might be that radiotherapy alone or in combination with idelalisib has led to the full remission of MCC [11].

## MATERIALS AND METHODS

### Patient samples

Twenty-one formalin fixed and paraffin embedded (FFPE) MCC tissues, 18 primary and 3 metastases, were obtained from the archives of the Department of Pathology, Maastricht University Medical Center +, Maastricht, the Netherlands. All use of tissue and patient data is in agreement with the Dutch Code of Conduct for Observational Research with Personal Data (2004) and Tissue (2001, “www.federa.org/sites/default/files/digital_version_first_part_code_of_conduct_in_uk_2011_12092012.pdf**”**). MCC have been defined by histology and immunohistochemistry for cytokeratin 20 (CK20), neural cell adhesion molecule (CD56), synaptophysin, chromogranin A and MCPyV (anti-large T antibody CM2B4, SANTA CRUZ BIOTECHNOLOGY) and have been reviewed by 2 experienced pathologists (VW, AZH).

### Cell lines

The MCPyV-positive MCC cell lines MKL-1, MKL-2, WaGa, PeTa; the MCPyV-negative MCC cell lines MCC13, MCC26 and the B-ALL cell line REH were used. All MCC cell lines were kindly provided by Jürgen Becker (University Hospital Essen, Essen, Germany). REH was obtained from the Leibniz Institute DSMZ-German Collection of Microorganisms and Cell Cultures. The multiple myeloma cell line U266 was kindly provided by Department of Transplantation Immunology, Tissue Typing Laboratory from the Maastricht Medical Center, NL. As described by Ikeda and colleagues U266 is negative for the PI3K p110δ subunit [14], and thus served as negative control in this study. The cell lines were cultured in Gibco^®^ RPMI 1640 medium (Life-science) with 10% fetal calf serum (FCS) (Gibco^®^, Thermo Fisher Scientific) in an incubator at 37° C and 5% CO_2_.

### Western blotting

The MCPyV-positive cell lines MKL-1, MKL-2, WaGa, PeTa and MCPyV-negative cell lines MCC13, MCC26 and the B-ALL cell line REH were grown in T25 flasks (25 cm^2^). The cells were harvested, pelleted, washed with cold PBS and resuspended in protein stabilizing buffer which consists of CHAPS Cell Extract Buffer (cell signaling technology) and phosphatase-protein inhibitor cocktail (Thermo Fisher Scientific). The proteins were isolated by freezing and thawing cycles by using liquid nitrogen. The protein concentration was determined by using the Pierce^™^ BCA Protein Assay Kit (Thermo Fisher Scientific) according to the manufacturer’s instructions. 35–60 µg protein samples were separated by SDS-PAGE in 12% (w/v) polyacrylamide gels, transferred to nitrocellulose membranes according to the manufacturer’s instructions using Bio-Rad Mini Protean tetra systems. Followed by blocking with 5% BSA (Sigma), incubated with primary antibodies anti-PI3K p100δ A-8 1:300 (SANTA CRUZ BIOTECHNOLOGY); PI3K p100α antibody 1:1000 (cell signaling technologies), AKT antibody 1:1000 (cell signaling technologies) and pAKT (Thr308) antibody 1:1000 (cell signaling technologies) and monoclonal anti-β-actin antibody clone AC-15 1:10000 (Sigmal-Aldrich) diluted in blocking buffer and incubated over night at 4° C, washed and incubated with the secondary antibody Alkaline Phosphatase AffiniPure F(ab')_2_ Fragment Goat Anti-Mouse/Rabbit IgG + IgM (H + L) (Jackson ImmunoResearch). The blots were developed by NBT/BCIP (Thermo Fisher Scientific) incubation according to the manufacturer’s instructions. The blots were scanned using Canon CanoScan 9000F Mark II.

### Immunofluorescence microscopy

The expression of PI3K p110δ was assessed by immunofluorescence microscopy (IFM) in 18 primary and 3 metastatic MCCs, MCPyV-positive MCC cell lines MKL-1, MKL-2, WaGa, PeTa and MCPyV-negative MCC cell lines MCC13 and MCC26. Further the expression of PI3K p110δ was analyzed in the B cell acute lymphoblastic leukemia (B-ALL) cell line REH. All samples were formalin fixed and paraffin embedded. The FFPE sections were deparaffinized and rehydrated in xylene and ethanol baths. The antigen retrieval was performed by boiling the slides in 1 mM EDTA pH 8.0. The slides were blocked in 5% BSA TBST, incubated with the first antibody anti-PI3K p100δ antibody A-8 (SANTA CRUZ BIOTECHNOLOGY) at 4° C over-night, followed by washing and incubation with the secondary antibody Goat anti-Mouse IgG (H + L) DyLight 488 (Thermo Fischer Scientific).The nuclei of the cells were stained with DAPI mounting medium (VECTASHIELD, VECTOR LABORATORIES) according manufacturer’s instructions. The detection of the fluorescence stained cells was performed with the Leica microscope DM 5000 B (Leica).

### Treatment of cell lines with PI3K inhibitors

The cell lines MKL-1, MKL-2, WaGa, PeTa, MCC13, MCC26 and REH were treated with idelalisib (Selleckchem, Germany) and BYL719 (Selleckchem, Germany). Both agents were dissolved in DMSO. Following concentrations were used for the treatments: 10 nM, 100 nM, 500 nM, 1 µM, 12.5 µM, 25 µM, 50 µM and 100 µM.

The cells were incubated in a 96 well plate (Greiner Bio-One, Austria) for 120 h, in Gibco^®^ RPMI 1640 medium (Life-science) with 10% fetal calf serum (FCS) (Gibco^®^, Thermo Fisher Scientific) in an incubator at 37° C and 5% CO_2_. The effect of the drug on the cell viability was assessed by the XTT assay (Thermo Fisher Scientific) according to the protocol provided by the manufacturer. The read out of the XTT assay was done with the iMark™ Microplate Absorbance Reader (BIO-RAD).

To determine the effect of idelalisib on the PI3K pathway the phosphorylation of AKT was assessed by Western blotting. Therefore, the cells were treated with 0.2% DMSO, 1 µM, 12.5 µM and 25 µM of idelalisib for 72 h.

### Statistics

The half maximal inhibitory concentration (IC_50_) values were determined with Graphpad Prism 6. By using the One-Way ANOVA test the significance of the dose response curves was determined. The western blots were quantified via ImageJ [24].

## CONCLUSIONS

The tested MCCs and MCC cell lines express PI3K p110δ. However, the MCC cell lines seem to be resistant towards treatment with the specific p110δ-inhibitor idelalisib. Our data indicate that idelalisib monotherapy will not suffice to treat MCC. In as much MCC patients might benefit of a combination of radiotherapy and idelalisib remains to be elucidated by clinical studies.

## SUPPLEMENTARY MATERIALS FIGURE


