# Supplementary material for: Phosphatidylinositol 3-kinase p110δ expression in Merkel cell carcinoma
Source: Oncotarget. 2018 Jul 3;9(51):29565–73. doi: 10.18632/oncotarget.25619 (PMC6049866; doi:10.18632/oncotarget.25619)
Supplement: Supplementary file 1 [file oncotarget-09-29565-s001.pdf]

Phosphatidylinositol 3-kinase p110δ expression in Merkel cell carcinoma

SUPPLEMENTARY MATERIALS

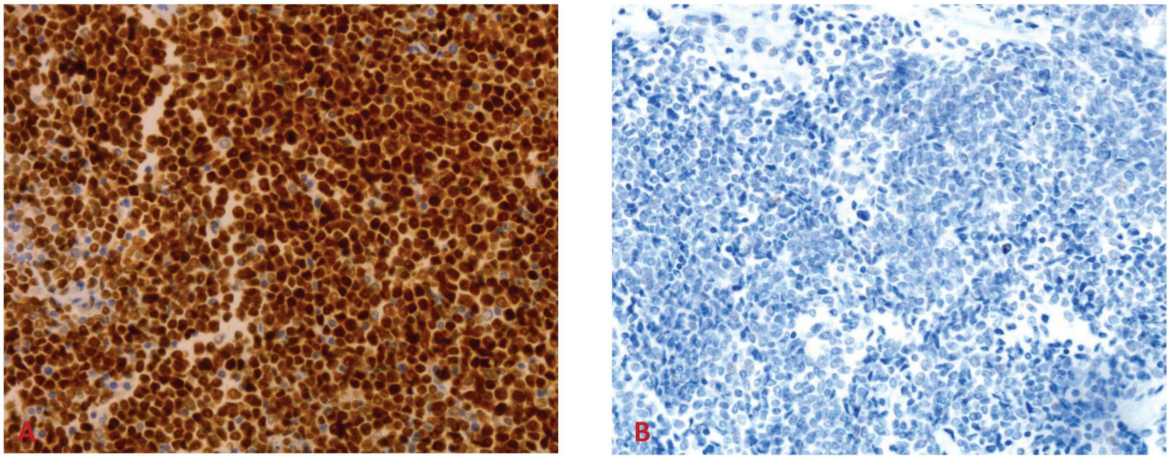

Supplementary Figure 1: MCPyV IHC of ID3 (**A**) and ID8 (**B**). ID1 MCC cells showing a specific and distinct nuclear expression of the MCPyV LT antigen (A) whereas, ID2 MCC cells were negative for MCPyV.
